# Swaging and Finishing Plates

**Published:** 1862-07

**Authors:** Ambler Tees


SWAGING AND FINISHING PLATES.—*By Ambler Tees.*—Each dentist having his own particular mode of manipulating, and perhaps habituated to that mode, it might appear like presumption, in the attempt of one to divert the other from his way. But having been conceded, and from the rapid and important improvements in dentistry of late years been proven, that an interchange of ideas is beneficial, we hope that we are not presuming in offering to the readers of the "Quarterly" our method of *swaging and finishing plates.* If it can be of any benefit to the "*young practitioner,*" whose education has given the worthy editors of other dental journals so much *concern*, so much *anxiety*, and so many *sleepless nights*, this effort, perhaps, may not be in vain; trusting that the "*old practitioner,*" whose education is completed, may discover something new, and also be edified.

In taking an impression for an atmospheric plate, to insure accuracy, care should be taken that it adheres to the roof of the mouth. To keep it perfect, and to remove it with more ease, the forefinger of each hand may be placed on each side of the jaw; and on raising the muscles, the impression will drop.

If the ridge of the jaw should present an acute angle, rendering the model difficult to mould, plaster should be run around it, (previously oiling to prevent it adhering) and then moulded. The plaster remaining in the sand will give a perfect impression of the ridge, and an excellent cast will be the result. By allowing the plaster to harden over night, bubbling will much better be prevented than by warming, altho' there is seldom any trouble from this source.

Where an atmospheric plate is required, there should invariably be two sets of dies; both male dies being made of pure block tin. One set should be kept for a finishing touch, since the little rugœ, on the first, becomes battered down, often rendering a suction impossible. Zinc is frequently used, but the shrinkage of it gives much trouble. The vacuum of partial sets on atmospheric principle should be always made on the roof of the mouth, allowing the plate to extend around at least four or five of the natural teeth. Sets, where the cavity is formed in the front part of the mouth, upon the rugœ, invariably prove failures; and the dentist finds that he is compelled to rely upon what perhaps his patient dreads, "*those horrid clasps.*"

In partial sets where no cavity is necessary, zinc will answer a better purpose, as only one set of dies will be requisite. In fitting clasps in such cases, a mistake is often made in getting the gold or silver, for the purpose, of too great thickness. If the tooth around which it is to be fitted is larger at the crown than at the neck, (which is often the case) a wide clasp should be made, of the same thickness as the plate, reaching from the neck to the crown. If the tooth is of the same thickness at the neck as at the crown, then a narrow clasp may be used about two numbers thicker than the plate.

In arranging the articulation for a whole set, in order to get the exact length of the teeth, the patient should be directed to fix the mouth in its natural position. The lip may then be lifted, and with a pointed instrument a line should be drawn on the wax, about one-sixteenth of an inch below the point of the lower lip, from one corner of the mouth to the other. This will give the length of the upper front teeth. If both upper and lower sets are being fitted, the wax on the upper may be trimmed off to the line above mentioned; more wax than is needed placed around the lower plate, put in and trimmed off until the mouth assumes its natural appearance. The center must then be marked off, and the wax on both plates joined together by means of a pointed instrument. They may then be removed, and the teeth are ready to be articulated.

Choosing the size and color of the teeth is of great importance, and unless the dentist exercises great care, and calls forth all his artistic attainments, he is apt to fail in making a perfect set of artificial dentures. Much in this respect may be learned by observing the different colors of the natural teeth, and the blending of them in different individuals; at the same time paying attention to the complexion of the person, the color of the eyes, etc. This will enable the dentist to judge, in a great measure, of the color of the natural teeth of the one for whom he is inserting artificial ones.

When the teeth are ready for backing or lining, it is an excellent time to prepare for finishing the plate; and I may here remark, that much trouble will be saved, the risk of warping the plate diminished, breaking or cracking the teeth avoided, besides having a better chance to give a perfect finish,—to polish the plate on the brush with oil and emery, or oil and pumice stone, removing all scratches and file marks, immediately after the plate is fitted in the mouth, (which should always be done) and before the bite is taken.

The backings of the teeth should be parallel, and as far as possible equi distant from each other, and finished off neatly. The metal should be a number thicker than the plate, and thick enough to allow of counter sinking well, and to be riveted firmly to the tooth. After riveting, the surplus platina of the pin should be filed off on a level with the lining, and the lining itself filed with a smooth file. This will cause the solder to flow evenly. The plate should be scratched where the backings meet it, causing the solder to attach itself firmly and quickly. The solder also should be scraped, and pieces cut about as long as the width of each lining, and one-sixteenth of an inch wide, to be put where the lining and plate meet. Pieces about one-sixteenth of an inch square should be put over each pin, previously wetting with borax ground to the consistency of cream. If single gum teeth are used, a neat and beautiful finish may be made by joining each backing with a narrow strip, running from its base one half its length, or to the point where the gum meets the tooth.

In thus taking a little care at this stage of the work, cracking teeth in soldering may be prevented, since the manipulator will not be compelled to keep the teeth heated up too long, and after it is soldered will have but little finishing to do. After boiling in sulphuric acid, a molar file and a scraper will, in a few minutes, render it ready for the oil-wheel. And as the plate has already been polished, the backings only will need brushing sufficient to remove the file scratches. After washing with castile soap and water, the finishing touch may be given with prepared chalk and a fine brush.—*Dental Quarterly.*

